# Interventional closure of artificial vascular anastomotic fistula after aortic replacement: a case report

**DOI:** 10.3389/fcvm.2025.1526798

**Published:** 2025-02-26

**Authors:** Qingwang Hou, Tongfeng Chen, Xiaohu Wang, Yipin Zhao, Chong Chen, Yuhao Liu

**Affiliations:** ^1^ Department of Cardiology, Henan University People’s Hospital, Henan Provincial People’s Hospital, Zhengzhou, China; ^2^ Department of Cardiology, Fuwai Central China Cardiovascular Hospital, Zhengzhou, China

**Keywords:** aortic dissection, anastomotic fistula, interventional occlusion therapy, structure, case report

## Abstract

Two years ago, the patient suffered from type A aortic dissection. As a result, partial aortic dissection artificial vascular replacement and partial aortic arch artificial vascular replacement were performed. Six months after the operation, an anastomotic fistula in the ascending aorta was detected, which subsequently progressed to chronic heart failure of New York Heart Association (NYHA grade) class III. After eliminating the operation - related contraindications, the patient successfully had the fistula occluded through transcatheter ascending aorta - right atrial fistula in our hospital. After the operation, no abnormal shunt was found, and the short - term treatment effect was satisfactory.

## Clinical data

**Figure 1:**
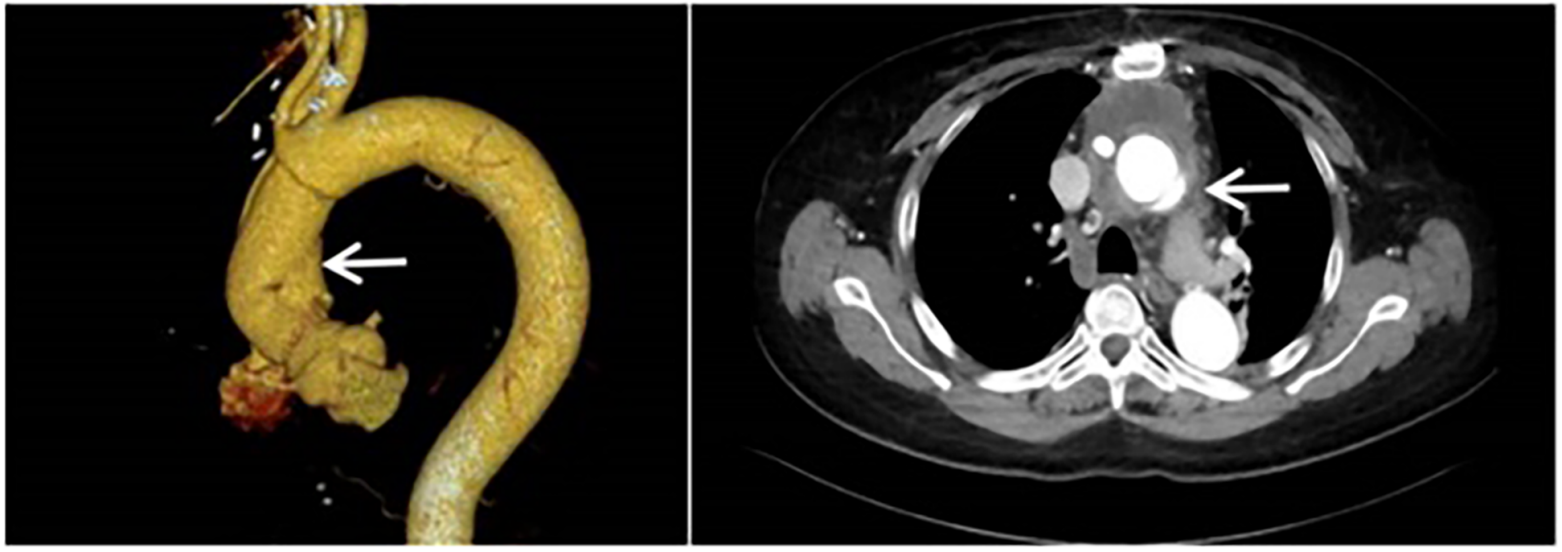
Computed tomography angiography of the aorta was performed when the patient was admitted to the hospital for the first time. (It was indicated that there was type A aortic dissection; the formation of true and false lumens could be seen distal to the origin of the right coronary artery in the ascending aorta).

The patient is a 61-year-old female who was admitted to the Structural Heart Disease Department of Fuwai Central China Cardiovascular Hospital on June 27, 2024 due to “chest tightness and shortness of breath after activity for more than 1 year.” She had a 20-year history of hypertension and her blood pressure was controlled between 130 and 145/75–95 mmHg with oral medications. Two years ago (in April 2022), due to type A aortic dissection, she underwent “partial resection of the ascending aorta with artificial vascular replacement and partial replacement of the aortic arch with artificial vascular” in our hospital emergently (Figure 1). A 28/8-mm Terumo single - branch artificial blood vessel was selected and wrapped with a pericardial patch. Meanwhile, aortic root wrapping and internal atrial drainage were carried out. Postoperative transesophageal echocardiography showed no aortic valve regurgitation, smooth blood flow in the artificial vessel, and no anastomotic leakage.

No special findings were observed during the re-examinations of color Doppler ultrasound and electrocardiogram at 1 month and 3 months after the operation. However, during the re-examination of color Doppler ultrasound 6 months after the operation (in October 2022), it showed an anastomotic fistula of the ascending aorta artificial vessel, while the blood flow in the artificial blood vessel of the aortic arch was smooth. No specific treatment was administered. In the recent years, the patient's chest tightness and shortness of breath aggravated, leading her to come to our hospital.

**Figure 2:**
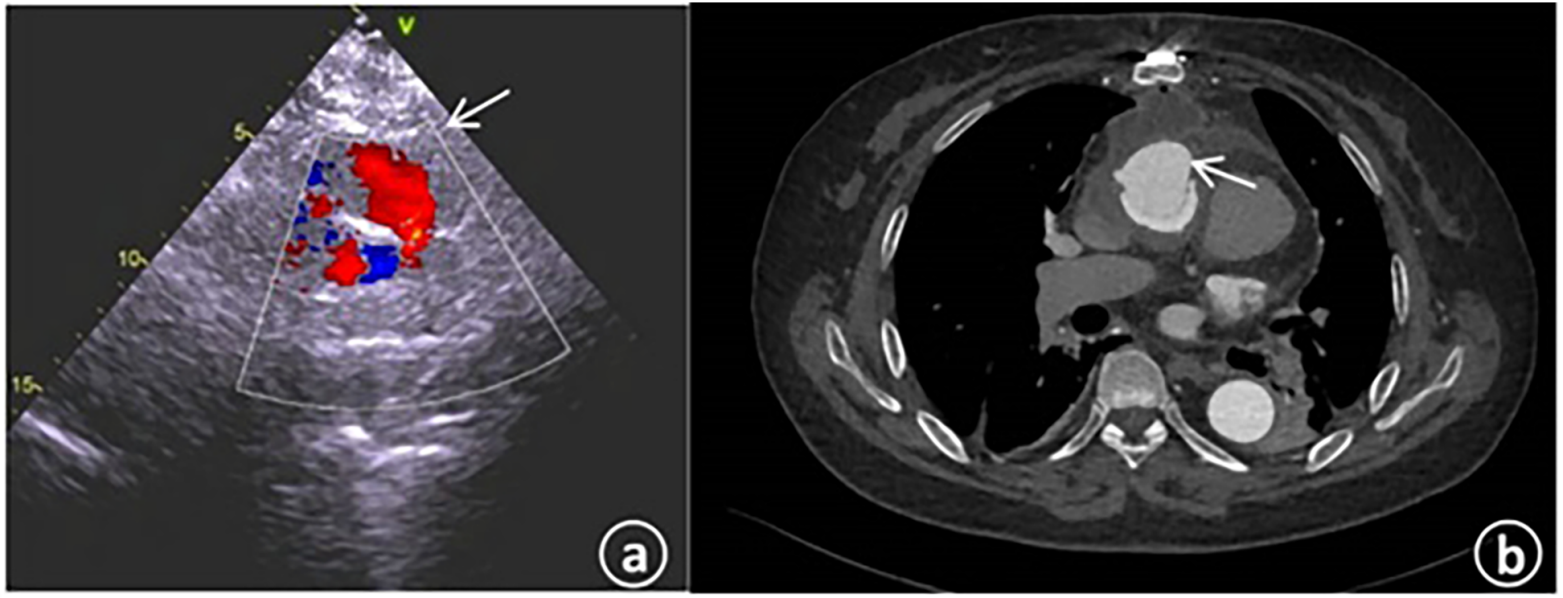
**(a)** The cardiac color – Doppler ultrasound upon admission revealed the presence of abnormal blood flow within the artificial blood vessel located on the posterior wall of the distal ascending aorta. **(b)** The computed tomography angiography of the aorta during admission indicated the existence of patchy contrast agent shadows beside the ascending aorta, with local communication to the right atrial appendage.

Physical examination results were as follows: Temperature was 36.8°C; pulse was 72 beats per minute; respiration was 16 breaths per minute; blood pressure was 130/72 mmHg; heart rate was 72 beats per minute with a regular rhythm. No murmurs were heard in each valve auscultation area. The breath sounds of both lungs were clear, and there were no obvious moist or dry rales. Laboratory tests indicated that there were no particular abnormalities in blood routine and biochemical indexes. The N-terminal pro-brain natriuretic peptide (NT-proBNP) was 484.8 pg/ml (with the normal range being 0–125 pg/ml). The electrocardiogram showed sinus rhythm, an increased negative value of Ptfv1, and T-wave changes in some leads. Transthoracic echocardiography revealed that the left ventricular end - diastolic diameter (LVEDd) was 53 mm; the left ventricular ejection fraction (LVEF) was 62%, and the systolic pressure of the pulmonary artery was approximately 33 mmHg. An abnormal blood flow with a width of about 3.3 mm was observed between the artificial blood vessel and the autologous blood vessel on the distal posterior wall of the ascending aorta (Figure 2a). An abnormal blood flow with a width of around 3.5 mm was seen around the artificial blood vessel on the proximal lateral wall of the ascending aorta, and the blood flowed into the right atrium. Aortic CT indicated that the contrast agent communicated with the right atrial appendage beside the ascending aorta artificial blood vessel (Figure 2b).

## Admission diagnosis

After ascending aorta replacement, anastomotic fistula of ascending aorta artificial blood vessel, cardiac insufficiency, grade III cardiac function (NYHA classification).

## Surgical procedure

Prior to the operation, the patient was thoroughly informed of all risks related to the surgery. The patient and her family consented to undergo interventional surgery and signed the informed consent form. On July 1, 2024, transcatheter occlusion of the fistula between the ascending aorta and the right atrium was performed under local anesthesia.

**Figure 3:**
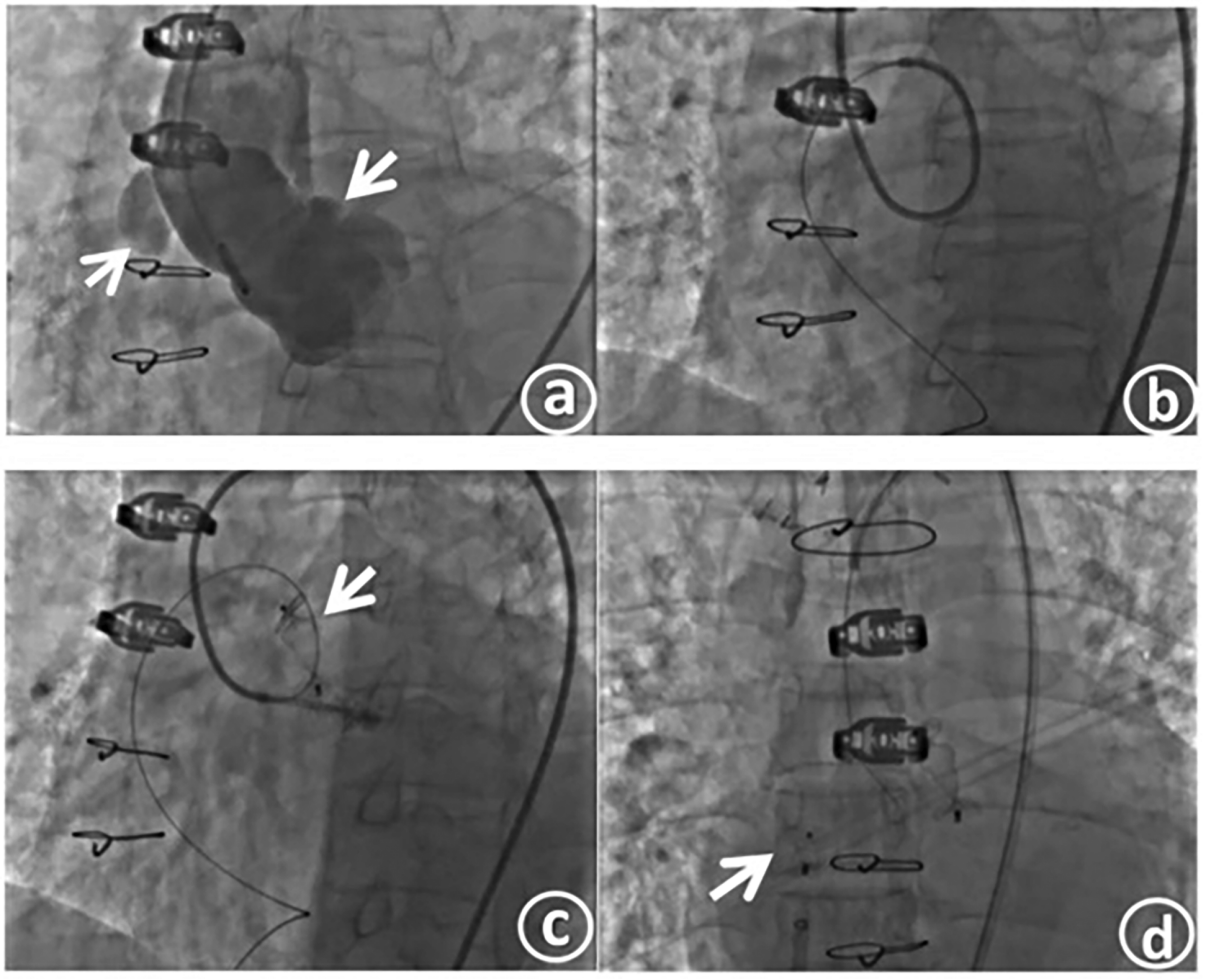
Aorticroot angiography shows an anastomotic fistula, with the contrast agent flowing into the right atrium **(a)**. The slippery guide wire traverses the fistula tract and is then delivered to the inferior vena cava through the outlet of the right atrium, thus establishing a complete track **(b)**. The occluder is released at the fistula opening on the aortic side **(c)**. The occluder is released at the fistula opening on the right atrial side **(d)**.

Under local anesthesia achieved with lidocaine, the right femoral artery and femoral vein were punctured, and catheter sheaths were inserted. A 6-F pigtail catheter was introduced for aortic angiography. The angiography results demonstrated: changes subsequent to aortic dissection surgery. Anastomotic leakage was identified in the anastomotic area between the distal end of the artificial blood vessel and the autologous aorta. The contrast agent infiltrated the aortic wrapping cavity through the leakage, forming a pseudoaneurysm, and then flowed into the right atrium (Figure 3a).

After confirming the location and morphology of the fistula in combination with the preoperative CT image, a 7 - F JL4 catheter was exchanged and inserted. Under the guidance of this catheter, a 0.035-inch, 260-cm super - smooth guidewire was passed through the leakage into the pseudoaneurysm body and then extended out to the inferior vena cava through the right atrial outlet, thereby establishing a complete track. Subsequently, the guiding catheter was advanced into the fistula tract (Figure 3b). The catheter was further maneuvered and passed across the anastomotic leakage into the pseudoaneurysm body. Contrast agent was injected through the catheter to measure the size of the fistula, which was approximately 7.1 mm × 3.6 mm.

After precisely identifying the location of the anastomotic leakage, a 12-mm type II vascular plug (Amplatzer vascular plug II, AVP II) was inserted through the angiography catheter and positioned at the leakage site. After confirming that the occluder was firmly fixed in place by the push - pull test, the occluder was released (Figure 3c). Aortic angiography was repeated, revealing successful occlusion of the anastomotic leakage, with only a small amount of contrast agent remaining in the pseudoaneurysm. A 6-F delivery sheath was selected and inserted through the right femoral vein, and a 6-F pigtail catheter was simultaneously introduced for right atrial angiography. After confirming the position, an MPA2 catheter was directly threaded through the right atrial fistula. Angiography indicated a fistula of approximately 4 mm. An 8-F delivery sheath was chosen, and a patent ductus arteriosus occluder (lifetech-PDA-8/10 mm) was selected and successfully used to occlude the fistula. After confirming the occlusion effect through angiography, the occluder was released (Figure 3d). Another round of angiography demonstrated the disappearance of the pseudoaneurysm.

## Post-operative re-examination

**Figure 4:**
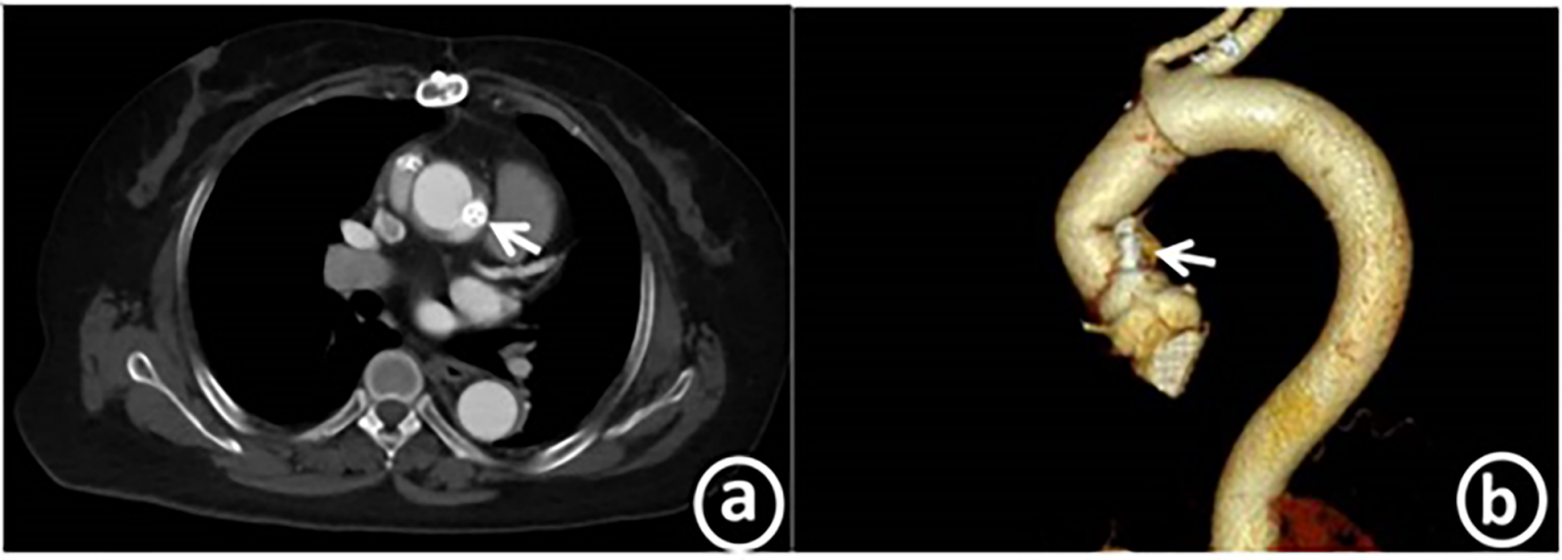
Post-operation, coronary CTA reveals the echo of the occluder, which is in a good position and has excellent molding. Panel a shows the axial plane, while panel b displays the curved planar reformation.

After the operation, the patient's symptoms were significantly alleviated, the cardiac function was at grade I (NYHA classification), and the N-terminal pro-brain natriuretic peptide level was 324.5 pg/ml. Transthoracic echocardiography indicated that the left ventricular end diastolic diameter was 52 mm, the left ventricular ejection fraction (LVEF) was 63%, and the systolic pressure of the pulmonary artery was approximately 26 mmHg. No obvious blood flow signal of the anastomotic fistula was detected. Aortic CT revealed the echo of the occluder with good molding, as presented in Figure 4.

Three months after the operation, the patient underwent a re-examination in the outpatient department of our hospital. The cardiac function remained at grade I (NYHA classification), and the N-terminal pro-brain natriuretic peptide was 127.6 pg/ml (with the normal range of 0–125 pg/ml). Transthoracic echocardiography showed that the left ventricular end-diastolic diameter was 52 mm, the LVEF was 68%, and the systolic pressure of the pulmonary artery was about 24 mmHg. No obvious blood flow signal of the anastomotic fistula was observed. Computed tomography angiography (CTA) demonstrated that the occluder was well - molded and no residual shunt was identified. The medium and long term efficacy still requires further follow up (Table 1).

**Table 1:** Comparison of the patient's reexamination indicators.

Patient	Admission	Postoperatively	Postoperatively (3 month)
HR (bpm)	72	78	70
BP (mmHg)	130/72	115/78	107/72
LVEF (%)	62	63	68
PASP (mmHg)	33	26	24
NT-proBNP (pg/ml)	484.8	324.5	127.6
NYHA classification	Grade III	Grade I	Grade I

HR, heart rate; BP, blood pressure; LVEF, left ventricular ejection fraction; PASP, pulmonary artery systolic pressure; NT-proBNP: N-terminal pro-brain natriuretic peptide.

## Discussion

Aortic root replacement surgery is a complex and arduous surgical procedure. Typically, during the operation, a channel between the aortic root and the right atrial appendage is reserved to drain the blood seeping from the anastomosis. Under normal conditions, this channel can close spontaneously and remain in a non - flowing state. However, if the degree of anastomotic leakage is significant, patients may exhibit obvious clinical symptoms such as dyspnea, fatigue, and even heart failure as they are unable to endure the continuous left - to - right shunt (1).

The presence of an aortic anastomotic fistula can lead to pseudoaneurysm, a patent false lumen, and even aortic rupture, all of which are associated with high mortality and morbidity rates. The occurrence of anastomotic fistula is not rare in clinical practice. In previous clinical scenarios, surgical repair has usually been regarded as the standard treatment for aortic anastomotic fistula (2). Re - thoracotomy entails a high risk of bleeding. Nevertheless, with sufficient preoperative preparations and the formulation of a rational treatment strategy, favorable treatment outcomes can be achieved clinically. However, due to the extensive surgical trauma and relatively high mortality rate of reoperation, which usually ranges from 13% to 41%, it is often not well - received by patients (3, 4). For surgeons, in patients undergoing reoperation, the adhesion between the artery, heart, and sternum is tight, resulting in dissection difficulties and a high bleeding risk. The risk of postoperative fistula infection is also substantial. Hence, the operation is demanding in terms of difficulty and technical requirements.

In recent years, transcatheter occlusion of aortic anastomotic fistula has gradually emerged as an effective alternative treatment option (4, 5). The postoperative effect is satisfactory, and it can alleviate the patient's symptoms with lower risks. In the 6 patients with interventional occlusion in Wu Wenhui's team (6), the postoperative shunt improved immediately, and the average hospital stay was merely 3 days. Moreover, there were no aortic and cardiac complications during the early and mid - term follow - up. Compared with surgical thoracotomy, interventional occlusion presents distinct advantages in multiple aspects.

Firstly, it is less invasive. The operation can be successfully completed under local anesthesia, reducing the patient's pain and postoperative recovery time. From an economic perspective, interventional occlusion is more cost - effective, which helps alleviate the economic burden on patients while ensuring the clinical treatment effect. However, interventional occlusion imposes high demands on the technical proficiency and experience of the surgical team, thereby limiting its widespread clinical application.

In this particular case, the patient was diagnosed with type I aortic anastomotic fistula (7). Intraoperative angiography revealed that the contrast agent flowed into the right atrium after being injected into the aortic wrapping cavity through the leakage. In light of this situation, the surgical team devised a double - occlusion surgical strategy for the inlet and outlet to minimize the occurrence of complications. The implementation of this surgical plan encounters several challenges. One of them is that the opening position of the aortic fistula is rather difficult. The scar tissue in the anastomotic fistula lesion area is tough and lacks elasticity. The fistula tract ascends along the aorta, making it arduous for the guidewire to reach the target position (8). Based on the preoperative CT examination and evaluation, the false lumen path is complex and irregular, significantly increasing the difficulty of guidewire passage and track establishment. To overcome these obstacles, the medical team successfully reached the fistula by leveraging the supporting force of the 7F JL4 catheter and sent a 260 cm loach guidewire, which smoothly entered the fistula tract. Through the femoral vein approach, the loach guidewire was successfully captured, and a complete track was established.

After the track was established, selecting the appropriate occluder became another crucial challenge. Due to the specific shape and structure of the fistula tract, which can be slit - like or elliptical, there is a lack of corresponding occluding devices in clinical practice. Through a comprehensive evaluation of the fistula tract morphology using preoperative CT images, the surgical team opted for a type II vascular plug occluder. By capitalizing on its small size and high flexibility, it can better conform to the anatomical structure of the fistula tract and minimize residual shunt. In combination with the intraoperative angiographic images, the surgical team finally placed a 12-mm type II vascular plug at the aortic fistula and selected an 8/10-mm patent ductus arteriosus occluder at the right atrial opening. The postoperative angiography results demonstrated no significant shunt, achieving an ideal outcome. The postoperative occlusion effect is excellent. The clinical symptoms improved rapidly after discharge. During the follow - up, there were no complications such as occluder displacement, enlarged leakage, hemolysis, or thrombosis.

In terms of long - term prognosis, since the occurrence of anastomotic leakage is somewhat related to the fragility of the vascular tissue at the anastomotic site, and there remains a possibility of residual fistula after occlusion, interventional occlusion is not a definitive solution. Active outpatient follow - up should be conducted, and medical attention should be sought promptly if clinical symptoms recur.

The technology of transcatheter closure of artificial vascular fistula has a high success rate and a low complication rate. The diameter of the postoperative aortic false lumen decreases rapidly, and the need for repeat aortic surgery is significantly reduced. It is an effective treatment method, yet it also entails potential risks. Firstly, issues such as occluder displacement, fistula enlargement, and aortic injury may arise during the operation. Additionally, if the occlusion is incomplete and residual shunt persists, and the pressure difference across the shunt channel is large, the fistula may further expand, thereby exacerbating the patient's condition (9). Therefore, meticulous preoperative evaluation and management of these risks are the crucial prerequisites for the surgical outcome. Combining previous surgical experience, our team discovered that after single - occluder occlusion, due to residual shunt, the fistula tract gradually expands, and the recurrence of anastomotic fistula may occur, further leading to cardiac insufficiency. Thus, the uniqueness of this case lies in the adoption of the double - occlusion strategy for the inlet and outlet, which not only guarantees the surgical effect but also effectively prevents the recurrence of anastomotic fistula, providing an effective treatment plan for patients with anastomotic fistula after aortic replacement.

Compared with surgical procedures, which have high requirements for both patients and surgeons, interventional occlusion provides a more suitable treatment option for this type of patients, and also offers an important reference for the treatment of similar cases in the future. However, more clinical cases and experience are required to further validate its clinical efficacy.

## Data Availability

The original contributions presented in the study are included in the article/Supplementary Material, further inquiries can be directed to the corresponding author.
